# Experimental transmission of the relapsing fever spirochete *Borrelia persica* in its tick vector *Ornithodoros tholozani* by transstadial, transovarial, and hyperparasitism routes with description of dynamics within the tick host

**DOI:** 10.1186/s13071-025-07093-3

**Published:** 2025-11-07

**Authors:** Gabriela Kleinerman, Yaarit Nachum-Biala, Reinhard K. Straubinger, Michael Ben-Yosef, Dor Shwartz, Gad Baneth

**Affiliations:** 1 Koret School of Veterinary Medicine, The Hebrew University, Rehovot, Israel; 2 Department of Bacteriology and Mycology, Institute for Infectious Diseases and Zoonoses, Ludwig-Maximilians-Universität München, Munich, Germany

**Keywords:** *Borrelia persica*, Relapsing fever, *Ornithodoros tholozani*, Argasidae, Transmission routes

## Abstract

**Background:**

*Borrelia persica* causes tick-borne relapsing fever, a potentially fatal human and animal disease, in the Middle East and Central Asia. The transmission of *B. persica* in *Ornithodoros tholozani* ticks has been reported to be transovarial, but little is known about the pathogen’s lifecycle in its vector and other pathways of transmission.

**Methods:**

To further understand the transmission of *B. persica*, colony-bred *O.* *tholozani* ticks were fed on blood in an animal-free system, followed during three lifecycles, and infected with cultured *B. persica*. Spirochetes were detected, and bacterial load was quantified by polymerase chain reaction (PCR).

**Results:**

*B. persica* colonization of the tick salivary glands was observed at week 4 post-infection of third-stage nymphs, a timeframe compatible with the duration required for the tick to become infective. Experimental *B. persica* transmission showed a decreasing rate of transstadial infection from 100% infection in larvae, to 55%, 20% and 25% in first-, second-, and third-stage nymphs, respectively, and 20% in adults. Five of the eight female ticks were PCR-positive for *B. persica*, and transovarial transmission was detected in the progeny of 3/5 infected females. Infection due to transovarial transmission was detected in 4% of the eggs laid by positive females, and in 2% of the larvae that originated from positive females. Transmission of *B. persica* between *O. tholozani* ticks was also experimentally shown to occur through hyperparasitism, when *Borrelia*-negative male ticks fed on infected third-stage nymphs.

**Conclusions:**

This study shows that *B. persica* can be maintained transstadially and transovarially in a tick colony, although transovarial transmission occurred at low rates. Additionally, transmission of *B. persica* was experimentally shown to occur through hyperparasitism between *O.* *tholozani* ticks, a transmission pathway that is likely to occur also under natural conditions. Finally, we determined that *B. persica* colonized the salivary glands within a period of 4 weeks, and thereafter the tick may be considered as ready to infect a host. Improved understanding of tick colonization by spirochetes and pathways of transmission described here will help to form concepts to disrupt the infectious cycle of tick-borne relapsing fever.

**Graphical abstract:**

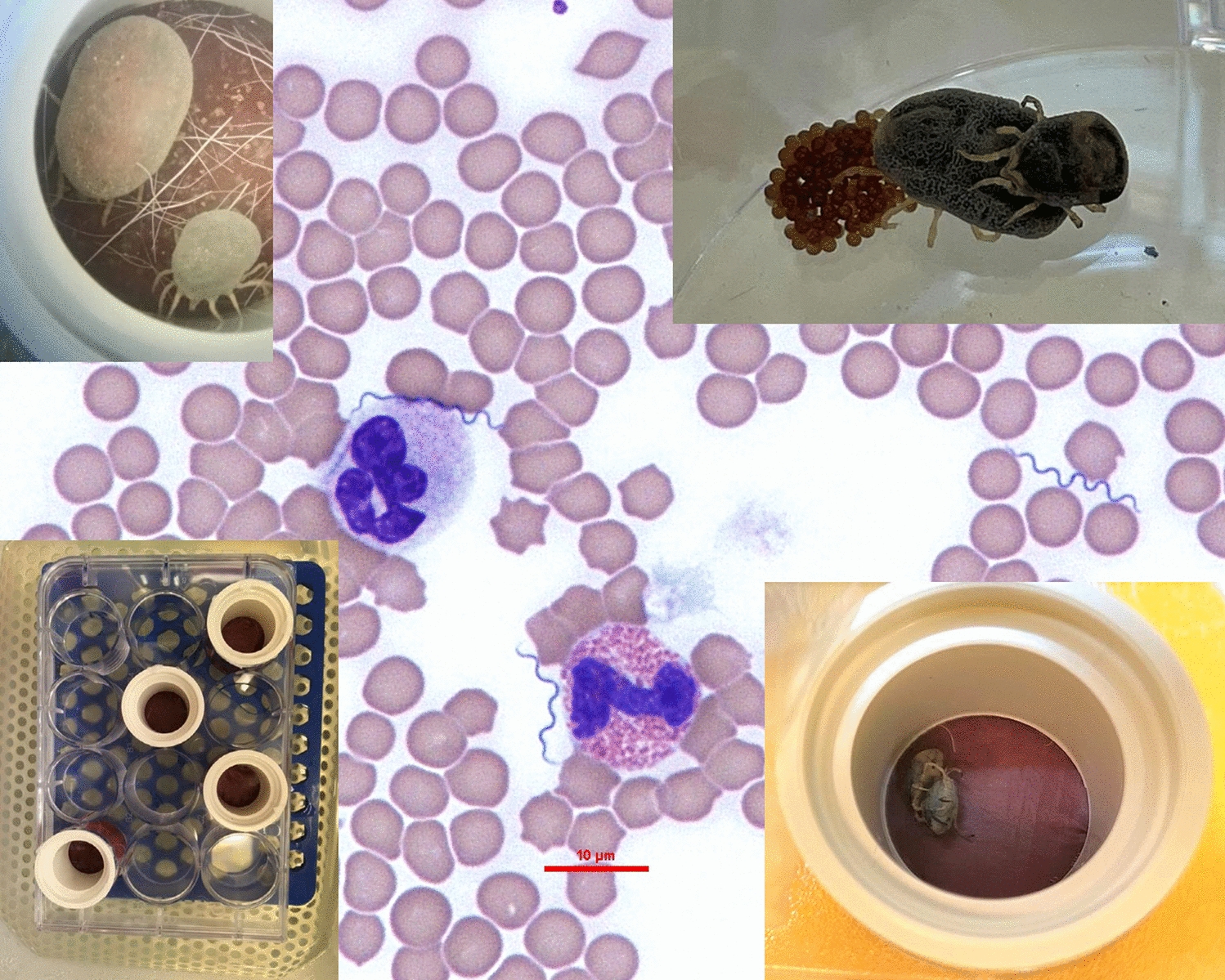

**Supplementary Information:**

The online version contains supplementary material available at 10.1186/s13071-025-07093-3.

## Background

Tick-borne relapsing fever (TBRF) is an acute infectious disease caused by infection with certain spirochete bacteria of the order Spirochaetales and the genus *Borrelia* [1]. The disease incubation period in humans in Israel is 2–12 days, followed by recurrent febrile episodes, chills, headache, myalgia, arthralgia, and abdominal pain. Fatal infections are associated with complications such as myocarditis, nephritis, liver failure, and cerebral hemorrhage [2]. *Borrelia persica*, which causes TBRF in parts of central Asia, the Middle East, and the eastern Mediterranean region, including Israel, is transmitted via the bite of the soft tick *Ornithodoros tholozani* [3].

*Ornithodoros tholozani* is prevalent in large parts of Asia, including India, Central Asian states such as Kazakhstan, Kyrgyzstan, Tajikistan, Turkmenistan, and Uzbekistan, Iran, Iraq, Syria, Jordan, Turkey, and Egypt in northern Africa [4]. The duration of the whole lifecycle of *O.* *tholozani* in nature lasts between 7 and 12 months and depends on host availability [4]. Its lifecycle includes one larval, three to four nymphal, and the imago stage (male and female) [5]. Blood feeding lasts between 20 min and 1 h, and all life stages feed on warm-blooded animals that enter their habitat [4]. Larvae and nymphs feed once in order to molt, and females usually feed before laying each batch of eggs, which may contain around 100 eggs. Males can feed several times in their lifetime [6]. The dynamics of infection with *B. persica* in its tick vector was reviewed by Felsenfeld as well as by Burgdorfer and Varma in the 1960s [5, 7]. Infection is transmitted through the tick bite. The time it takes from the acquisition of an infectious blood meal until the tick is capable of transmitting *B. persica* to the next host may vary among tick stages and has been reported to be up to 2 months [8]. However, the duration of the passage of *B. persica* from entry to the tick’s digestive system to reaching the salivary glands and further transmission to the tick’s host has not been studied. The different transmission pathways of *B. persica* within its tick vector and between ticks are not described in detail. It has been reported that transovarially infected *O. tholozani* were able to infect guinea pigs in all of their life stages except for the larval stage [9]. In another experimental study with *B. persica* in *O. tholozani*, the transovarial transmission rate increased from 11% to 47% from the first generation of infected females to the seventh generation; however, there was a significant loss of spirochete virulence in the guinea pig model [10]. In a recently published study, we showed that only 3 out of 787 larvae (0.04%) collected from caves in Israel were positive for *B. persica* when tested with a PCR protocol, and the infection rates in larvae were significantly lower than in nymphs, males, and females; showing that transovarial transmission under natural conditions is minimal [11].

Beside the transovarial route, pathogens can be transmitted between ticks through several other ways: by co-feeding, a pathway in which pathogens are transmitted from tick to tick via the vertebrates’ tissue independent of a systemic infection [12, 13]; transstadially, where the pathogens remain in the same tick and are passed on from one life stage to the next of the tick [14]; by ingestion of the tick by the vertebrate host as in *Hepatozoon canis* [15]; sexually during tick mating [16]; and lastly by hyperparasitism, a behavior by which ticks feed on other tick individuals of the same species [17]. Hyperparasitism can potentially be associated with direct transmission of relapsing fever borreliae between ticks and was reported to support transmission of *Borrelia crocidurae* and *Borrelia hermsii* transmitted by the soft ticks *Ornithodoros erraticus* and *Ornithodoros hermsi*, respectively [17, 18]. Hyperparasitism was also observed in *O.* *tholozani* [19], but no transmission experiment with *B. persica* was performed.

**Fig. 1:**
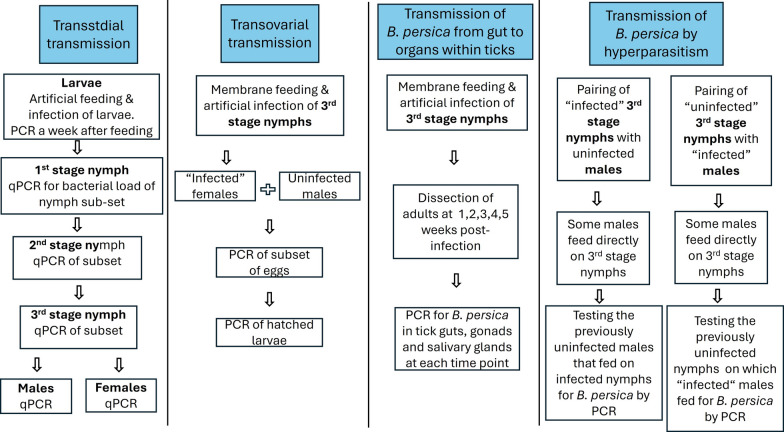
Flowchart of the different experiments included in the study

To better understand the transmission and dynamics of infection of *B. persica* in its tick vector, we developed an artificial feeding system and established a *Borrelia* spp.-free *O.* *tholozani* tick colony. We then infected *O. tholozani* with *B. persica* in an animal-free system, determined the time needed for *B. persica* to reach the salivary glands after the acquisition of an infected blood meal, and analyzed the rates of transstadial and transvoarial transmission in *O. tholozani*, as well as the transmission of *B. persica* by hyperparasitism (Fig. 1).

## Methods

### Establishment of an *O. tholozani* colony

A parent stock of adult *O. tholozani* was collected in a cave in the Ayalon Canada Park in central Israel (31° 49′ 39.5″ N, 35° 01′ 07.6″ E). Ticks were collected as described previously using a CO_2_ trap [4]. Briefly, three collector traps, each connected to a cool box emitting CO₂ from dry ice, were buried in the soil and left overnight. Ticks were identified morphologically as described by Filippova [20]. After oviposition, all males (*n* = 6) and females (*n* = 5) from the parent stock (generation zero) were analyzed by real-time PCR targeting the *Borrelia flaB* gene [21] for the possibility of infection and possible vertical transmission of *B. persica* organisms. All ticks used in the parent stock were found to be negative for *B*. *persica*. All tick stages were maintained under controlled conditions of 25 °C, 80% relative humidity, and darkness. Humidity was maintained by pouring potassium dihydrogen phosphate (KH₂PO₄) salt on a Petri dish and adding a small amount of water as described previously [22]. Larvae were maintained post-hatching in groups of five in 1.5-ml conical microcentrifuge tubes (Labcon, USA), with two holes in the lid and a permeable plaster (3M Micropore^®^) placed internally to prevent the escape of the ticks. Engorged larvae and nymphs of different stages were maintained individually under the same conditions. Engorged, pre-oviposition females were placed individually with one or two engorged males for mating in separate wells of a six-well plate (Number 3516, Corning, USA), with a small piece of paper to absorb secretions. After the first oviposition, males were removed and placed individually in tubes. Eggs were collected in groups of five and placed in tubes for hatching, and the females were left for at least 1 month to continue the oviposition.

### Artificial tick feeding

Larvae of *O. tholozani* were apparently too small to artificially attach and feed through a membrane. We therefore attempted to feed them directly by immersing them in blood. Infection of *Ixodes scapularis* and *O. hermsi* larvae with *Borrelia burgdorferi* and *B. hermsii*, respectively, by immersion in liquid suspension of these spirochetes was achieved previously in two studies [23, 24]. However, to our best knowledge, immersion with blood has not been performed to feed larvae of ixodid or argasid ticks previously. Feeding of larvae in our study was performed successfully in groups of five individuals, which hatched from eggs of females from the parent stock. Larvae were fed 14–32 days after hatching, by placing them directly in 1.5-ml conical microcentrifuge tubes (Labcon, USA) with 100 µl of fresh heparinized bovine blood (maximum 1-week-old) and leaving them overnight in a water bath at 35 °C. The next day, the blood was retrieved, and the larvae were washed three times with phosphate-buffered saline (PBS), dried on wipe paper, and then placed individually in a tube for maintenance and molting to first-stage nymphs.

Nymphs of all stages and adult ticks were fed in vitro through an artificial feeding unit adapted and modified from Krober and Guerin [25]. Ticks were fed in groups of five individuals of the same category (first-, second-, third-, and fourth-stage nymph, males and females) placed in 2-cm-long plastic tubes with 1.5-cm-diameter Corning^®^ Costar^®^ Netwell inserts (Corning, USA) where the mesh bottom had been removed, and closed at one end with Parafilm^®^ (0.13 mm thickness, Pecheney Plastics Packaging, USA). The inner surface of the film (in contact with the ticks) was applied with lipid extract of dog hair to stimulate attachment and feeding. The lipid hair extract was prepared following a procedure described previously [25] with some modifications. One gram of hair from a dog that was not treated with ectoparasiticides against ticks was dissolved in 40 ml of a chloroform/methanol mix (ratio of 2:1). The liquid was mixed for 30 min, decanted on a clean glass, and left overnight to evaporate the solvents. The dry material obtained was redissolved in 3 ml of the chloroform/methanol mix, the solvent was allowed to evaporate for 15–30 min on a hot plate at 40 °C, and the dry material obtained was subsequently applied to the Parafilm^®^ membrane with a soft brush. The tubes were placed membrane side down in a 12-well plate (number 3513, Corning, USA), and the wells were prefilled with 1.5 ml of heparinized bovine blood. Care was taken to ensure close contact between the membrane and the blood. Ticks were placed for feeding inside the tube, and the upper end of each tube was then sealed with Parafilm^®^ to prevent tick escape during feeding. The plate was then closed with its lid and placed in a warm bath at 37 °C for 1 h. To improve the attachment and engorgement of first-stage nymphs, the Parafilm^®^ membrane was perforated with a 23-G needle, allowing the ticks direct contact with the blood. After engorgement with blood, each tick was placed individually in a 1.5-ml conical microcentrifuge tube for molting. Single engorged males and females were placed together in wells of six-well plates for mating.

Prior to use for tick feeding, each bovine blood batch was analyzed for *B. persica* infection by real-time PCR targeting the *Borrelia flaB* gene [21] to verify that the blood meal was not infected.

### *Borrelia persica* culture

For this study, 100 µl of *B. persica* isolate (strain LMU-C01 passage 5, isolated from an Israeli cat) was cultivated in Pettenkofer/LMU Bp medium as described previously [26]. The cultures were incubated at 37 °C for 7 days until they arrived at their exponential phase. Viable *B. persica* spirochetes were counted under dark-field microscope in a Neubauer chamber of 0.02 mm depth. Spirochete suspensions were adjusted to the required concentration by dilution with fresh Pettenkofer/LMU Bp medium.

### *Borrelia persica* survival in bovine blood

Bovine blood, which was kept at 4 °C for 3 days, was prewarmed to 37 °C and then inoculated with 1.0 × 10⁶ *B. persica* spirochetes grown in culture, per ml of blood. The inoculated blood was kept at 37 °C and examined using dark-field microscopy for motility of spirochetes every hour for the first 24 h and then daily until no motile spirochetes could be observed. The purpose of this experiment was to evaluate how long dormant bovine blood would sustain the activity of *B. persica* and estimate whether the spirochete might survive longer within a fed tick only owing to the presence of bovine blood.

### *Borrelia persica* acquisition by ticks at defined time points

*Borrelia persica* from the in vitro-grown cultures were centrifuged and mixed with heparinized bovine blood, which was verified to be negative for *Borrelia* by PCR, to reach a final concentration of 1.0 × 10⁶ spirochetes per ml of blood, following a previous publication [27]. Infection of third-stage nymphs from a stock of colony-bred *B. persica* negative ticks raised in our laboratory was performed in the same way as the feeding of nymphs described above, with the addition of a 1-mm layer of sterile cotton placed inside the well within the *B. persica* infected blood, to prevent sedimentation of spirochetes during the infection. *O. tholozani* third-stage nymphs were allowed to feed for 1 h in a water bath set at 37 °C. After engorgement, the nymphs were removed, dried, and placed individually under maintenance conditions. Forty-two nymphs were fed with infected blood, and once they were engorged, they were divided into four groups of eight and one group of ten individuals, according to the time point of analysis post-infection of the ticks: 1, 2, 3, 4, and 5 weeks. For analysis at these time points, ticks were dissected as described previously [28]. Once the dorsal cuticle was separated, guts, salivary glands, and gonads were removed and placed in a droplet of sterile 1× Dulbecco’s PBS (Lonza Group, Basel, Switzerland). For DNA extraction, each organ (salivary gland, gonads, and guts) was placed in 50 μl of 1× PBS, then 180 μl ATL buffer (composed of sodium dodecyl sulfate, Qiagen, Dusseldorf, Germany) were added together with 20 μl proteinase K and left for incubation for 1 h at 56 °C. DNA extraction was carried out using the Qiagen DNeasy Blood and Tissue Kit (Qiagen, Düsseldorf, Germany) following the manufacturer’s protocol. Ten uninfected ticks were fed with noninfected blood and analyzed weekly from week 1 to 5 as controls, two ticks at each time point.

### Transstadial and transovarial transmission of *B. persica* in ticks

To study transstadial transmission, *O. tholozani* larvae from a stock of colony-bred *B. persica*-negative ticks raised in our laboratory were infected by immersion in 100 µl blood mixed with 1.2 × 10⁷ spirochetes per ml. The spirochete concentration to infect *O. tholozani* larva was chosen following a previous publication [24].

Preparation of the spirochete mixture included centrifugation of *B. persica* culture for 10 min at 5000 × *g* at room temperature to equally disperse the bacteria in the medium, and then mixing with heparinized bovine blood verified to be *Borrelia*-free by PCR to a final volume of 100 µl. Infection of larvae was performed by immersion, in the same way as the feeding of larvae described above. Five larvae were placed together in tubes filled with 100 µl of the mix of blood and *B. persica* culture medium at the concentration mentioned above, and left overnight in a bath set at 37 °C. Then, this mixture was removed, the larvae were washed three times with PBS, dried, and placed individually in maintenance tubes. To study the acquisition of *B. persica* by the larvae, 20 larvae were analyzed a week post-infection by real-time PCR targeting the *Borrelia flaB* gene [21], and their bacterial loads were quantified. The rest of the larvae were left to molt to first-stage nymphs, and these were further fed on uninfected blood using the artificial feeding system, to molt to successive stages. Twenty first-, second-, and third-stage nymphs, and 20 adults were analyzed to evaluate the presence of *B. persica* and quantify the bacterial loads in order to determine the transstadial passage of *B. persica* between the different tick life stages. For DNA extraction from whole ticks, ticks were washed individually with PBS and air-dried for 15 min on paper. The ticks were then separately sliced into small pieces with a sterile scalpel blade and manually crushed and homogenized in a tube with 180 ATL buffer using plastic microtube pestles for 1 min. DNA was then extracted using a commercial kit (DNeasy Blood & Tissue Kit, Qiagen) following the manufacturer’s protocol. Four microliters of DNA was used in each PCR reaction.

Transovarial transmission was evaluated by infecting eight third-stage nymphs with 10⁶ spirochetes per ml of blood and allowing them to molt to the adult stage. After molting, the eight females were individually paired with eight uninfected males for mating. Analysis of transovarial transmission of *B. persica* was done on 20 eggs and 20 larvae laid and hatched from each potentially infected female. The adults were dissected 50–60 days after oviposition, and each organ, including the salivary glands, the guts, and the gonads, was analyzed separately using real-time PCR targeting the *Borrelia flaB* gene [21]. DNA extraction from eggs was done on individual eggs as described previously [29]. Single eggs were placed separately in a 0.2 ml microtube and crushed with a sterile needle. Each microtube was then filled with 12 µl of extraction buffer containing 1 mg/ml proteinase K (Sigma-Aldrich, USA), 0.01 M NaCl, 0.1 M EDTA, 0.01 M Tris–HCl (pH 8.0), and 0.5% Nonidet P-40 substitute (Sigma-Aldrich, USA). Following spinning down of the tubes, they were incubated at 65 °C for 15 min, followed by inactivation at 95 °C for 1 min.

DNA extraction of larvae was performed from a single whole unfed larva, whereas DNA of adult tick organs extraction was performed individually for each organ, both using the Qiagen DNeasy Blood & Tissue kit (Qiagen). In addition, five uninfected females were paired with five uninfected males as a control group to compare the number of eggs laid and the rate of larvae hatching between infected and noninfected females.

### Spirochete acquisition and horizontal transmission between ticks

Hyperparasitism was analyzed by feeding ten previously unfed third-stage nymphs with blood mixed with 1.0 × 10⁶ *B. persica* spirochetes per ml of blood through a Parafilm^®^ membrane as described above and pairing them immediately after engorgement, individually with ten unfed uninfected males. Each pair of nymph and male was kept together in a separate tube for 3 h. This was done to study spirochete acquisition by the unfed male tick from the previously fed third-stage nymph. Immediately after the pairing ended, nymphs were searched for external lesions suggesting laceration caused by male mouthparts by viewing under a stereomicroscope (Stemi 2000-C, Zeiss, Jena, Germany). In another experiment, 12 engorged uninfected third-stage nymphs were paired with 12 unfed potentially infected males, which were infected at the third nymphal stage individually, and kept together in separate tubes for 3 h, to study possible spirochete transmission from the adult potentially infected males to the engorged third-stage nymph. Here too, lesions on the nymphs, suggesting laceration caused by the male’s mouthparts, were searched for immediately after pairing ended. The ability of the ticks to horizontally acquire and transmit the spirochetes by hyperparasitism was examined directly 5 weeks after pairing by extracting DNA from the third-stage nymphs, which had molted to adults, and from males from both trials and performing *flaB* real-time PCR of individual salivary glands, guts, and gonads of these ticks [21].

### *Borrelia persica* detection and molecular analysis

*Borrelia persica* infection in tick and blood DNA samples was confirmed by real-time PCR targeting a 346-bp fragment of the *flaB* gene, using primers Fbpbu (GCTGAAGAGCTTGGAATGCAACC) and Fbpcr (TGATCAGTTATCATTCTAATAGCA) [21]. Real-time PCR was carried out in 20 μl Maxima Hot Start PCR Master Mix (Thermo Scientific, Loughborough, UK) with 0.25 μM of each primer and 0.6 μl syto9 (Invitrogen, CA). The reaction was carried out at an annealing temperature of 54 °C and 60 °C for ticks and bovine blood samples, respectively, and 50 cycles followed by a melting phase. Plasmids (Topo Ta cloning Kit, Life Technologies, Grand Island, USA) containing a *B. persica flaB* gene insert and DNA from a *Borrelia*-negative tick were employed as positive and negative controls, respectively. All samples that were positive were sequenced using the Sanger technique after being purified using a PCR purification kit (Exo-SAP, NEB; New England Biolabs, Inc. Ipswich, MA). PCR products were sequenced using both forward and reverse primers using the BigDye Terminator v3.1 Cycle Sequencing Kit and an ABI PRISM 3100 Genetic Analyzer (Applied Biosystems, Foster City, CA, USA), at the Center for Genomic Technologies, Hebrew University of Jerusalem, Israel. Forward and reverse sequences were trimmed for low quality (average quality of 10 bases exceeding 15) using the Chromas version 2.6.6 software then aligned and assembled using MEGA software version 10. Both forward and reverse primers sequences were removed from the final sequence. The sequences were further compared with sequences from GenBank using the BLAST algorithm [30].

### *Borrelia persica* quantification

Quantitative PCR of *Borrelia* DNA was carried out on ticks positive for *B. persica* by real-time PCR by using a quantitative real-time PCR (qPCR) amplifying a 106-bp segment of the *flaB* gene following a previous publication [31]. A standard curve was generated to assess the detection limit of the qPCR using tenfold dilutions of a plasmid containing a *B. persica flaB* 512-base-pair insert with dilutions from 50 to 1.0 × 10⁸ copies of *flaB*. A second standard curve was made to control the DNA content of tick DNA by dilutions of a second plasmid containing a tick *16S rRNA* 480-base-pair insert containing from 100 to 1.0 × 10⁹ copies of the gene to assess the detection limit of the qPCR. The following formula was used for the calculation of the initial copy number of the plasmid: number of gene copies calculated as [(amount in ng/μl × 6.022 × 1023)/length of plasmid × 1 × 109 × 650] (Genomics and Sequencing Center, University of Rhode Island, Kingston, USA [32]). Standard curves were obtained by running triplicates of the plasmid dilutions with *flaB*- or *16S rRNA*-insert reference points and plotting their Ct values against the log of *B. persica flaB* or tick *16S rRNA* plasmids using the StepOne software version 2.2.2 (Thermo Fisher Scientific). The qPCR of *flaB* amplification was performed as described before [31]. qPCR amplification of a 216-bp segment of the tick *16S rRNA* was done using primers F126 (TTTTGGGACAAGAAGACCCTATG) and R344 (CAACATCGAGGTCGCAAACTA) designed for this study. The qPCR was carried out with an initial hold for 4 min at 95 °C, followed by 40 cycles of 15 s at 95 °C, 30 s at 62 °C and a melting phase which started at 60 °C, each step rising by 0.1 °C, and ended at 95 °C with a hold for 90 s at the first step and 5 s at the subsequent steps. For tick *16S rRNA* real-time PCR, the reaction was performed in 20 μl containing 1 μl of plasmid DNA, 0.25 μM of each primer, 0.6 μl of syto9 (Invitrogen), 10 μl of Maxima Hot Start PCR Master Mix (Thermo Scientific), and DNA-free water added to obtain a final volume of 20 μl. DNA samples from the *B. persica* tick-positive samples (4 and 2 μl, for the *flaB* and *16S rRNA* reactions, respectively) were run by qPCR together with the plasmid dilutions in duplicates with negative and nontemplate controls (NTC). The actual loads of *B. persica* were calculated using StepOne software version 2.2.2 and were expressed as *Borrelia flaB* copies per 10^5^ copies of tick *16S rRNA* gene. Samples that did not reach a minimum threshold of 50 *flaB* copies were considered negative.

### Statistical analysis

Bacterial loads in the different tick life stages and in the tick guts at different timepoints were compared by the nonparametric Kruskal–Wallis test for *k* number of independent samples using the SPSS software (IBM, NY, U.S.A). The Mann–Whitney test for two independent variables was used to compare spirochete loads in the salivary glands and in the guts using the SPSS software. Larvae hatching rates from infected and noninfected females were compared by the chi-squared test using the SPSS software.

## Results

### Artificial feeding and lifecycle completion

The whole lifecycle of *O. tholozani* ticks was completed three times in consecutive experiments using the artificial feeding system in this study. In total, 5788 ticks of different life stages were tested, including 1605 larvae, 601 first-stage, 364 second-stage, and 269 third-stage nymphs, 30 females and 34 males, and 2885 eggs. The first cycle was completed in 351 days (standard error, SE = 17.35), the second cycle in 546 days (SE = 13.8), and the third cycle in 238 days (SE = 6.15) (Additional file 1: Additional Table 1). The difference between the lengths of the cycles was due to a technical reason related to the feeding system. In the second cycle, we changed the dog from which we produced the lipid hair extract since the dog used in the first cycle was no longer available. The time between the end of one lifecycle and the next, i.e., from the first oviposition of a certain tick generation until the feeding of the first batch of larvae of the next generation, ranged between 45 and 63 days [average (AV): 51.5, SE = 5.7].

**Table 1:** *Borrelia persica* acquisition by third-stage nymphs at defined time points post infection. Presence of *B. persica* DNA and quantification of *B. persica* loads in tick organs at different time points post infection

Weeks post-infection	Total number of ticks	Life stage analyzed	No. of *B. persica flaB*-positive ticks	Infected organ	Rate of infection (%)	Average *B. persica flaB* copies per 10^5^ tick *16S* gene copies in guts (number of guts quantified)	SE	Average *B. persica flaB* copies per 10^5^ tick *16S* gene copies in SG (number of SG quantified)	SE
1	8	N3	8	Guts	100	67.4 (8)	42.0		
2	8	N3	8	Guts	100	4.7 (8)	1.9		
3	8	N3	7	Guts	90	5.3 (6)	5.1		
4	8	5 N3, 2 F, 1 M	6	5 Guts and 4 SG	80	48.7 (4)	48.4	0.5 (3)	0.15
5	10	6 N3, 3 N4, 1 M	5	5 Guts, and 1 SG	50	28.5 (5)	22.9	30.4 (1)	

*N3* third-stage nymph, *N4* fourth-stage nymph, *F* female, *M* male, *SG* salivary glands, *SE* standard error

Reproductive capacity parameters, engorgement, and molting rates were measured for the different tick generations (Additional file 1, Table 1). The average number of eggs laid per female was 56.8, 183.7, 184.2, and 189.0, for the parent stock, first, second, and third generation, respectively. The percentage of larvae hatching was 60.2%, 91%, and 94% for the first, second, and third generations, respectively. The period of days between the female tick engorgement and oviposition was 44.2, 21.2, 28.7, and 26.4 for the parent stock, first, second, and third generation, respectively. Average percentage rates of engorgement were 81.9% (SE = 5.1), 58.0% (SE = 10.3), and 68.2% (SE = 2.3) for the first, second, and third generation, respectively; average molting rates were 56.9% (SE = 11), 85.6% (SE = 3.5), and 85.5% (SE = 3.9), for the first, second, and third generation, respectively. The average mortality rates of ticks in all three lifecycles were 7.1% (SE = 3.1), 4.5% (SE = 2.3), 2.4% (SE = 2.4), 12.2% (SE = 3.9), and 0%, for larvae, first-, second-, and third-stage nymph, and adults, respectively. Females were fed only once in their adult life prior to oviposition. Females, except for those of the parent stock and of the third generation, which were analyzed after the first oviposition, were allowed to continue with additional cycles of folliculogenesis for up to 5 months. Out of nine females belonging to the first and second generations, two females had four ovipositions, two had three ovipositions, three had two ovipositions, and two had only one oviposition. The other tick life stages were also fed only once in each life stage prior to molting, except for an additional feeding attempt that was carried out for those ticks that refused to feed in the first try, which was done a week after the first one. This part of the study showed that the whole lifecycle of *O. tholozani* can be reproduced under laboratory conditions exclusively by artificial feeding with no need for feeding on experimental animal hosts.

### *Borrelia persica* survival in bovine blood

Follow-up of spirochete motility in the bovine blood was continued over 8 days. Motile spirochetes were observed until 96 h post-inoculation and not thereafter, supporting the idea that *B. persica* requires being in a tick or vertebrate host to continue its long-term survival.

### *Borrelia persica* acquisition by third-stage nymphs at defined time points post-infection

Ticks raised in the laboratory, which originated from the parent stock, were brought from the egg stage to the third-stage nymphs by artificial feeding on uninfected bovine blood. At that stage, 42 third-stage nymphs were fed with heparinized bovine blood containing 10⁶ *B. persica* spirochetes per ml, and were divided into five groups according to the time point of analysis post-infection. The groups included ticks examined by PCR for *B. persica* in their guts and salivary glands, 1, 2, 3, 4, and 5 weeks after infection (Table 1). All 16 (100%) of the third-stage nymphs tested at 1 and 2 weeks after infection were found to be positive for *B. persica* by PCR only in their guts. At week 3, seven out of eight (87.5%) ticks tested were positive for *B. persica* in their guts, and one was negative in both guts and salivary glands. In the fourth week, two out of eight third-stage nymphs had molted to females and one to a male, while the remaining five stayed as third-stage nymphs. Six (75%) of these ticks were infected, including five third-stage nymphs and one female. When relating to all tick stages, *B. persica* DNA was found in the guts of four ticks and salivary glands from three. Two third-stage nymphs were positive in both their guts and salivary glands, two were positive only in their guts, and one was positive only in its salivary gland. The positive female was infected in its guts and its salivary glands. Of the ten ticks evaluated after 5 weeks of infection, three nymphs had molted to fourth-stage nymphs, and one had molted to a male. Infection was detected in five ticks, including five guts and one salivary gland. Three third-stage nymphs were positive only in their guts, one fourth-stage nymph was infected in its gut, and another fourth-stage nymph was infected in its gut and salivary glands. The male was negative.

In total, out of the 42 infected third-stage nymphs in all the groups, 34 (81%) ticks (31 third-stage nymphs, two fourth-stage nymphs, and one female) were positive for *B. persica* by the *flaB* PCR, including 33 guts and 5 salivary glands, with infection of salivary glands detected only in ticks analyzed at week 4 and 5 after infection (Table 1). The average *B. persica* loads in the guts, expressed as *Borrelia flaB* copies per tick *16SrRNA* gene copy, ranged between 4.7 and 67.4 in the different weeks (Table 1). The copy number of *B. persica* in the guts at week 1 (AV = 67.4) was significantly higher than at week 3 (AV = 5.5) and 4 (AV = 48.7) (Kruskal–Wallis test *H* = 8.25, *df* = 3, *P* = 0.041; Table 1). However, when comparing the *B. persica* loads in the guts of ticks at week 5 of infection (AV = 28.5) with those found at the other time points, only a nonsignificant trend was observed (Kruskal–Wallis test *H* = 8.79, *df* = 4, *P* = 0.066). The average *B. persica* loads in the salivary glands at weeks 4 and 5 post-infection were 7.9 *flaB*-copies per tick *16SrRNA* gene copies (SE = 7.5), and in the guts analyzed at week 4 and 5 it was 37.46 (SE = 23.43) with no significant differences between loads in the salivary glands and in the guts with those found at the other time points (Mann–Whitney *U*-test: *U*_(13)_ = 16.00, *Z* = −0.309, *P* = 0.825). The results described in this section portray the dynamics of *B. persica* within experimentally infected ticks, with movement of infection from the tick gut to the salivary glands noted from 4 weeks post-infection.

### Transstadial and transovarial transmission of *B. persica*

Transstadial transmission was analyzed by infecting 161 larvae with 1.2 × 10⁷ *B. persica* spirochetes per ml of blood and testing 20 individuals from each tick life stage, e.g., larvae, first-, second-, and third-stage nymphs, and adult ticks (ten females and ten males). Larvae were tested 1 week after feeding on infected blood, whereas first-, second-, and third-stage nymphs were analyzed a week after molting; adults were analyzed 2 weeks after molting. Except for larvae, which were analyzed after engorgement, the remaining stages were analyzed unengorged. The rate of infection decreased from 100% (20/20, 95% CI 83.16–100%) in larvae to 55% (11/20, 95% CI 31.53–76.94%), 20% (4/20, 95% CI 5.73–43.66%), and 25% (5/20, 95% CI 8.66–49.10%) in first-, second- and third-stage nymphs, respectively. Adult ticks showed an infection rate of 20% (4/20, three male ticks and one female tick).

Average *B. persica* loads in ticks were 243.8 (SE = 42.1), 21.6 (SE = 7.7), 38.1 (SE = 4.9), and 61.2 (SE = 21.9) *flaB*-copies per tick *16SrRNA* gene copies in larvae and first-, second-, and third-stage nymphs, respectively. Average *B. persica* loads in adults were 199.2 *flaB*-copies per tick *16SrRNA* gene (SE = 25.7) [244.4 (SE = 50.2) and 63.5 in males and females, respectively] (Table 2). There was a significant difference in parasite load between tick stages (Kruskal–Wallis test *H* = 20.18, *df* = 4, *P* < 0.001). Post hoc pairwise comparisons using Dunn’s test with Bonferroni correction indicated that the larvae had a significantly higher parasite load than first-stage nymphs (*Z* = 18.344, *P* = 0.002), but no other pairwise differences were significant.

**Table 2:** Transstadial transmission of *Borrelia persica* infected at the larval stage

Tick life stage	Total number of ticks	Number of *B. persica flaB*-positive ticks	Rate of infection (%)	Average *B. persica* *flaB* copies per 10^5^ tick *16S* gene copies (number of ticks quantified)	Standard error
Larvae	20	20	100	243.8 (20)	42.2
N1	20	11	55	21.6 (9)	7.7
N2	20	4	20	38.1 (3)	4.9
N3	20	5	25	61.2 (3)	21.9
Adults	20	4	20	199.2 (4)	25.7
Males	10	3	30	244.4 (3)	50.2
Females	10	1	10	63.5 (1)	

Presence of *B. persica* infection and quantified loads in subsequent stages

Transovarial transmission was tested by mating eight infected females with eight uninfected males and analyzing 20 eggs and 20 larvae laid and hatched from each of the eight potentially infected females. Because only eight eggs were analyzed from one of the females, a total of 148 eggs and 160 larvae were analyzed. The number of days from feeding and engorgement of the females to the beginning of the first oviposition ranged between 21 and 44 days (average = 26.6, SE = 2.6), and the number of eggs laid per female ranged between 70 and 190 (average = 129.6, SE = 13). Larval hatching rate ranged between 67% and 85% (average = 77%, SE = 2.3). Dissection and molecular analysis of the females and males, which were infected at third-stage nymph, was done after the first oviposition between 12 and 16 weeks post-infection, and real-time PCR targeting the *Borrelia flaB* gene [21] was done on the salivary glands, guts, and gonads. Of the eight females, five were infected (62.5%). Three of them were positive in their guts, five in their salivary glands, and three in their ovaries. Of those, three females were positive in their guts, salivary glands, and ovaries, and two females only in their salivary glands. All eight uninfected males that were mated with the females were negative for *B. persica* DNA. Regarding *B. persica* infection in eggs, 4 of 148 eggs (2.7%) analyzed were positive by PCR. The positive eggs belonged to three positive females. Considering only the eggs laid by infected females (*n* = 5), the rate of egg infection was 4 of 100 (4%), ranging from 5% to 10% per infected female (Table 3). Regarding *B. persica* infection in larvae that hatched from the eggs of the experimentally infected ticks, 2 of 160 larvae tested were positive (1.3%), and both belonged to larvae hatched from eggs laid by one positive female. Considering only larvae derived from positive females, the rate of larvae infection was 2 of 100 (2%) and 10% per the single positive female, which gave rise to the infected larvae (Table 3).

**Table 3:** Transovarial transmission of *Borrelia persica* to eggs and larvae from infected *Ornithodoros tholozani* female ticks

Infected female identifying number	Total eggs laid	Total eggs analyzed	Total larvae hatched (%)	Total larvae analyzed	*B. persica flaB*-positive organs in the female	*B. persica flaB*-positive eggs (%)	*B. persica flaB*-positive larvae (%)
776	148	8	116 (83)	20	All organs negative	0	0
805	106	20	62 (72)	20	SG, guts, and ovaries	0	0
756	126	20	71 (67)	20	SG, guts, and ovaries	1 (5)	0
911	156	20	116 (85)	20	SG	0	0
779	70	20	37 (74)	20	All organs negative	0	0
716	190	20	127 (75)	20	SG, guts, and ovaries	1 (5)	0
916	137	20	87 (74)	20	SG	2 (10)	2 (10)
766	104	20	70 (83)	20	All organs negative	0	0

*SG* salivary glands

Five uninfected females were paired with five uninfected males as controls. The period of days from feeding and engorgement of the adults until the beginning of the first oviposition ranged between 21 and 29 days (AV = 26.4, SE = 1.6), and the number of eggs laid per female ranged between 84 and 263 (AV = 189, SE = 29.3). The larvae hatching rate ranged between 74% and 94% (AV = 88%, SE = 3.9%). The hatching rate of the larvae originating from the control group of uninfected females (88%) was close to being significantly higher than the hatching rate of the larvae derived from *B. persica*-infected females (75%) (Welch’s t-test, t(5.477) = −2.49, *P* = 0.051).

The outcomes of the experiments on transstadial and transovarial transmission indicated that both modes of *B. persica* transmission could be demonstrated by experimental infection using artificial tick feeding, and that the rate of transovarial transmission was considerably lower than that of its transstadial counterpart.

### Horizontal transmission of *B. persica* between ticks by hyperparasitism

**Fig. 2:**
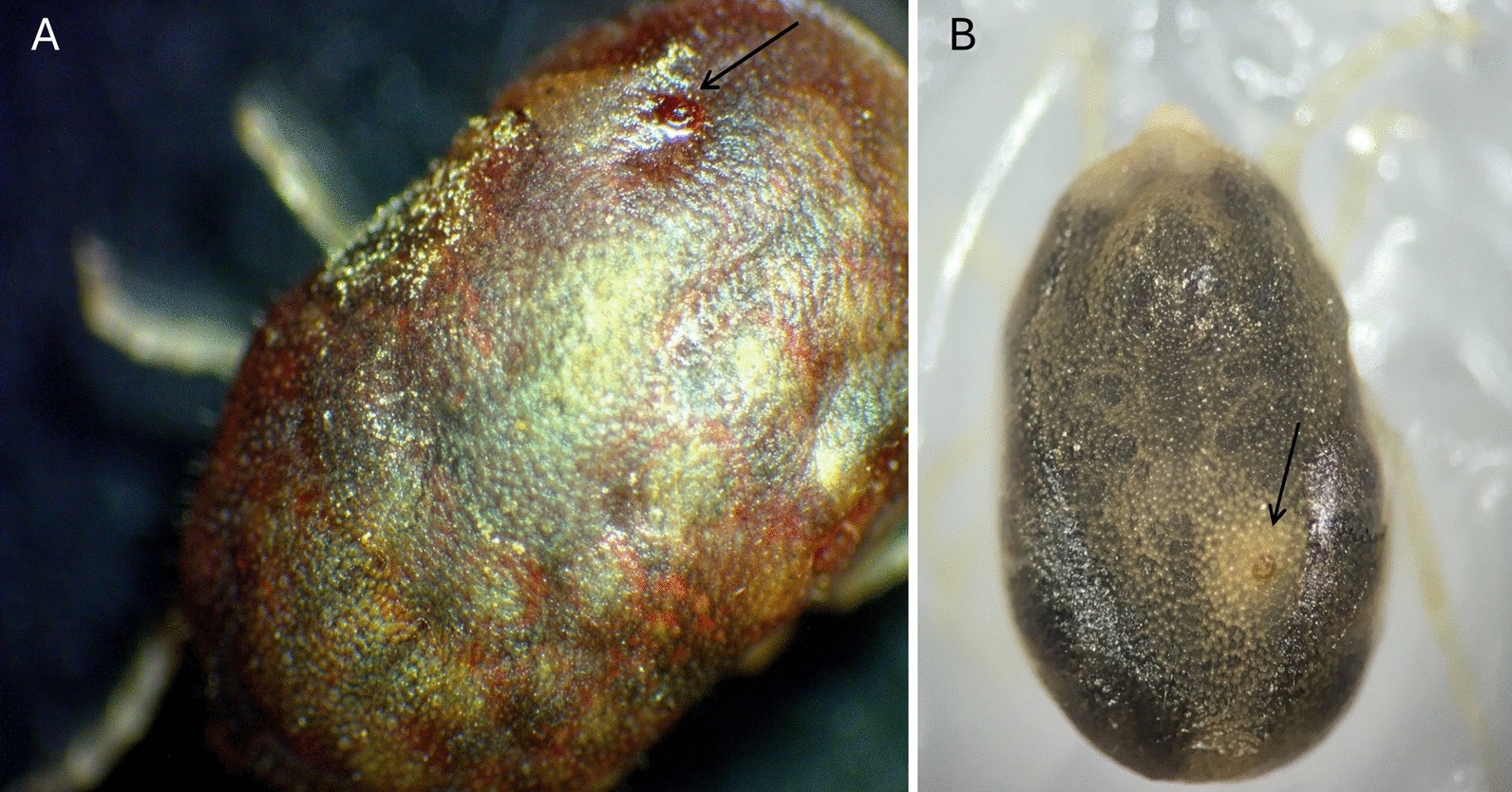
Laceration lesion in a nymphal dorsal plate (black arrow) immediately after hyperparasitism by a male (**A**), and healed laceration lesion in the dorsal plate (black arrow) of the same nymph 5 weeks after the breach caused by a male (**B**)

Twenty-two third-stage nymphs infected with heparinized bovine blood containing 10⁶ spirochetes per ml were divided into two groups. In the first group, each infected third-stage nymph was individually paired immediately after infection with an uninfected unfed male for 3 h to study possible direct acquisition of *B. persica* by the males from the nymphs. Biting of nymphs and engorgement of the males with laceration of nymph dorsal plates caused by male mouthparts were observed in three nymph-male pairs (Figs. 2A, B). Five weeks after pairing, three out of ten potentially infected and engorged nymphs molted into fourth-stage nymphs, one molted into a male, and the rest remained as third-stage nymphs. Molecular screening by real-time PCR targeting the *flaB* gene [21] revealed that five out of these ten ticks were positive for *B. persica*, including three third-stage and one fourth-stage nymph. All positive ticks showed infection in the guts, and one fourth-stage nymph showed *B. persica* DNA also in the salivary glands. Out of ten uninfected, unfed males before the pairing, three showed blood in their gut during their dissection, which was performed 5 weeks after pairing with the nymphs. These three males had been paired with third-stage nymphs, which showed laceration lesions in their dorsal plates after the pairing, and two of these males were positive for *B. persica flaB* DNA in their guts at week 5 of the experiment. These two positive males had been paired 5 weeks earlier with two nymphs that were PCR-positive, demonstrating transmission of *B. persica* by hyperparasitism. The third engorged male, which was negative by PCR, was paired with a nymph that turned out to be PCR-negative for *B. persica* when tested.

In the second group, 12 uninfected, engorged third-stage nymphs were paired individually immediately after feeding with 12 potentially infected unfed males for 3 h. After the pairing, five nymphs showed laceration of the dorsal plates and one on the ventral exoskeleton, caused by male mouthparts. Five weeks after pairing, 3 out of 12 engorged uninfected third-stage nymphs molted into males, 1 into a female and 1 into a fourth-stage nymph, and the remaining 7 remained as third-stage nymphs. *Borrelia*-specific *flaB* DNA real-time PCR screening revealed that all of the previously engorged uninfected ticks were negative for *B. persica* DNA, while two of the potentially infected males, which fed on the uninfected ticks, harbored *B. persica* DNA in their guts. In essence, *B. persica* was not transmitted during hyperparasitism from infected males to engorged nymphs. The results of this part of the study indicate that *B. persica* can be transmitted between *O. tholozani* ticks by feeding directly on each other.

### Molecular analysis

All positive samples were sequenced. In total, 101 *flaB* DNA sequences of *B. persica* were obtained. From these, 38 sequences were from 38 ticks infected during the transstadial infection experiment, 38 sequences were from positive organs of 34 ticks infected weekly during 5 weeks, 17 sequences were from positive organs and offspring of infected females during the transovarial infection experiment, and 8 sequences were from organs of infected nymphs and males that participated in the hyperparasitism infection experiment. All DNA sequences were 100% identical to each other. Four DNA sequences were submitted to GenBank (accession nos. MW284983-86). Uninfected control ticks of all life stages and all DNA extractions from negative controls were PCR-negative for *B. persica*.

## Discussion

This study demonstrated and followed the dynamics of *B. persica* transmission by the transstadial and transovarial modes, as well as horizontally from tick to tick through hyperparasitism. Three lifecycles of the soft tick *O. tholozani* were completed in vitro by feeding the ticks exclusively in an artificial system using heparinized bovine blood. To our knowledge, this is the first time that an artificial feeding model without contact of *O. tholozani* ticks to warm-blooded hosts has been successfully established. It proves that this tick species can be maintained throughout its lifecycle in the laboratory using only artificial feeding and overcoming the problem of feeding larvae, which do not feed readily through artificial membranes. Other methods used to feed *O. tholozani* were proven to be efficient, such as feeding through rat or rabbit skin and Baudruche membrane made of ox intestine. However, only nymphs and adult ticks were able to feed through a membrane, while the younger larval stage fed on live rats [6, 33]. Pathogen transmission studies using artificial feeding of ticks have advantages and disadvantages over animal models [34]. Membrane feeding of ticks enables controlled infection with a set concentration of pathogen under uniform, stable conditions. It is also more ethically acceptable, as it does not require the use of laboratory animals, allows infection of large numbers of ticks simultaneously, and is usually less expensive than running experiments with rodents or other mammals such as rabbits, dogs, or ruminants. On the other hand, using experimental animal hosts mimics natural infection in a better way, with the involvement of the host immune responses and other host–pathogen interactions, such as the effect of the tick’s saliva on transmission. The present study used an artificial feeding system because of its ethical advantage and the opportunity to produce large numbers of ticks in stable and easily repeatable conditions.

We were able to infect larvae and third-stage nymphs of *O. tholozani* with a predefined number of *B. persica* organisms. We clearly show that larvae can be infected with *B. persica* through feeding by immersion in infected blood, and these further transmit the spirochete through the successive tick life stages, although with an initially decreasing infection rate. In addition, we found that migration and arrival of *B. persica* from the tick’s gut to the salivary glands takes between 3 and 4 weeks from ingestion of the infected blood meal. *Borrelia persica* remains in the gut after its dissemination to the salivary glands, presumably serving as a reservoir of spirochetes to repopulate the salivary glands after the next blood meal. Similar results were obtained for *B. hermsii* infection in *O. hermsi*, where the dissemination and establishment of the spirochete infection in the salivary glands took about 3 weeks [35]. In an experimental infection of *Ornithodoros turicata* with *Borrelia turicatae*, the colonization of spirochetes in the salivary glands was observed after molting from third-stage to fourth-stage nymphs, which took 4 weeks [36]. In the present study, the gut contents of *B. persica* started with high loads in the first week and then decreased through the following weeks, although after week 5, no significant differences in spirochetal load in the gut were observed compared with the remaining time period.

The transstadial transmission study showed a decreasing infection rate from 100% in larvae, to 55%, 20%, and 25% in first-, second-, and third-stage nymphs, respectively. Adult ticks showed a 20% infection rate. Significantly higher *B. persica* loads were observed in larvae compared with the rest of the tick’s life stages (*P* < 0.001). The large number of *B. persica* organisms in the larvae does not necessarily indicate that a high number of replicating *B. persica* is present, since the larvae were analyzed 1 week after having an infected blood meal. The high level of spirochetes in the infected larvae shows that all tested individuals successfully ingested a large number of *B. persica* from the blood meals, which remained in their guts at the time of PCR. The rest of the tick life stages were analyzed after molting to their next stage; therefore, the number of *B. persica* determined probably reflects the potential infective loads that remained after molting.

Five out of eight females infected as third-stage nymphs had *B. persica* DNA in their organs between 12 and 16 weeks post-infection when the transovarial transmission mode was studied. Three of these five positive female ticks were able to transmit *B. persica* to their progeny. Considering only the laid eggs and the emerged larvae that originated from infected females, the rate of egg and larvae infection was 4% (ranging from 5% to 10% per individual transmitting female) and 2% (10% per transmitting female), respectively. Three *B. persica*-positive females contained infected ovaries; two of them laid infected eggs. The remaining positive females were found to be infected in their salivary glands only, but despite this, only two eggs and two larvae that originated from one of these salivary gland-positive females were positive for *B. persica* when tested with our PCR protocol. It should be considered that all analyses were performed after the first oviposition, and it is unclear whether *B. persica* colonized immature follicles during the next folliculogenesis after oviposition, and if this could have affected the presence and the load of *B. persica* in the ovary.

Both Adler et al. and Pavlovskiy and Skrynnik were able to demonstrate transovarial transmission of *B. persica* in *O. tholozani* to its progeny [9, 37]. Pavlovskiy and Skrynnik also found that the infection may be maintained for two generations of ticks, but the rate of transmission was not described in that study, and the larvae used for the experiments were unable to infect guinea pigs [9]. Transovarial transmission of *B. persica* was also demonstrated by Balashov in a study where the transmission rate increased from 11% to 47% from the first generation of infected females to the seventh generation. However, there was a significant loss of the spirochetes’ virulence when they were transmitted to guinea pigs by eighth-generation ticks [10]. A main difference regarding transovarial transmission between those older studies and our current study was that animals were used as sources for infection and also as blood sources in the previous studies, while we used *B. persica* cultures to infect ticks and fed them only artificially. Whether artificial feeding results in loss of virulence, as was found in the study using guinea pigs [10], remains to be determined in future studies. The differences in transovarial transmission rates between those studies may be due to the different techniques used and the lack of live animal involvement in our study. However, the results of our previous study on naturally infected ticks collected in caves in Israel showed that only 0.04% of the unfed larvae were found infected, while 4.4% of the males, 3% of the females, and 3.2% of the nymphs trapped in natural habitats were infected with *B. persica* [11]. This finding supports the idea that the low rate of transovarial transmission found in our experimental model is also valid for the natural settings.

Other RF *Borrelia* were found to have higher transovarial transmission rates in various studies than *B. persica*. *Ixodes scapularis* transmitted *B. miyamotoi* transovarially, ranging from 6% to 73% in its progeny [38]. In another study, the transovarial transmission rate of *B. miyamotoi* was 90.9% and the mean filial infection prevalence of the resulting larval clutches was 84.4%, with rates that varied from 36% to 100% [39]. A third study of transovarial transmission of *B. miyamotoi* in *I. scapularis* showed that the filial infection rate of larval batches from females infected as larvae or nymphs ranged from 3.3% to 100% with a median rate of 71%, and that a lower filial infection rate was associated with lower maternal spirochete loads [40]. *Borrelia duttoni* showed very variable transovarial transmission rates of 2% to 98% in the progeny of infected *Ornithodoros moubata* females, and its infection was maintained over 3 years in successive stages of the tick by transovarial passage only. Nonetheless, a notable decrease in its virulence to mice was observed [7]. In the case of *B. hermsii*, transovarial transmission in *O. hermsi* is rare, therefore, most larvae of *O. hermsi* collected in nature were not infected [35]. *B. turicatae* was maintained transstadially through six nymphal stages of the tick *O. turicata* until molting to adults [41], and it was also transovarially transmitted through eggs to first-stage nymphs, which were shown to be infective to mice, monkeys, and humans [42].

Owing to the long duration of spirochete maintenance shown in several RF tick vectors, such as *O.* *hermsi*, *O. turicata*, and *O. tholozani*, these tick vectors may be considered as reservoirs for the respective RF *Borrelia* spp. transmitted by them [9, 33, 43]. However, our present experimental study and previous field study [11] found relatively low rates of transovarial transmission of *B. persica* in *O. tholozani*. Taking this finding into consideration, the hypothesis suggesting that *O. tholozani* acts as the main reservoir of TBRF in nature [4, 9] should be reconsidered, and an animal reservoir is likely of major importance for the maintenance of infection in nature. This agrees with our previous study, which associated the presence of a blood meal and specifically certain hosts’ blood meals with *B. persica* infection of *O. tholozani* in nature, and complements this with the presence of infection in compatible wild mammal hosts in a wildlife animal study [11].

Another possible mode of *B. persica* transmission evaluated in this study was hyperparasitism, which is a horizontal, direct way of transference of *B. persica* between ticks. Hyperparasitism was previously observed in uninfected *O. tholozani* under laboratory conditions [19]. In our study, hyperparasitism was detected in three pairs of ticks by observing attachment and seeing lesions of laceration of the nymphal dorsal plates, with *B. persica* transmission and its acquisition detected in two males that fed directly on infected third-stage nymphs. This is the first time that a horizontal passage of *B. persica* between ticks through this behavior has been demonstrated. Although it is not known whether this mode of transmission occurs under field conditions, hyperparasitism is likely to take place also in populations of ticks in natural settings. It may act as an additional important mechanism for *B. persica* spread within a local tick population in environments such as soil of caves and other locations where ticks are found at high densities. Therefore, ticks acquiring *B. persica* by feeding on infected warm-blooded hosts may later give rise to additional infected ticks, thus increasing the likelihood of disease transmission to vertebrate hosts.

The fitness consequences of *B. persica* infection in *O. tholozani* were observed when we compared hatching rates of larvae originating from infected and uninfected females. Although the overall hatching rate of larvae from uninfected females was close to being significantly higher than in those derived from infected females (*P* = 0.051), future research should compare additional fitness parameters of infected and uninfected ticks throughout the entire lifecycle to fully understand whether *B. persica* infection is associated with negative or positive effects on the development of their tick vector and further in its transmission to a susceptible host.

## Conclusions

This study shows that, following the ingestion of an infectious blood meal, *B. persica* colonizes the tick’s salivary glands within 3–4 weeks. Thereafter, the tick may be considered infectious for a warm-blooded host. *B. persica* can be maintained transstadially and transovarially in tick colonies, although at low infection rates. Therefore, the role of the tick as the main reservoir of the bacterium in nature is questionable, whereas transmission from an infected animal reservoir appears to be a major mode of transmission when combining the results of the present study with those of our research on natural infection [11]. This study has also revealed the possibility of direct transmission of *B. persica* from tick to tick by hyperparasitism, which could be an additional important mode of its transmission and maintenance in tick populations.

## Supplementary Information

Additional file 1.

## Data Availability

All data acquired in this study are available publicly. DNA sequences generated in this study were deposited in GenBank as accessions MW284983–MW284986 for *B. persica flaB* partial sequences.

## Abbreviations

CI: Confidence interval
NTC: Non-template controls
PBS: Phosphate buffered saline
PCR: Polymerase chain reaction
TBRF: Tick-borne relapsing fever
